# Prevalence, Associate Health Conditions and Evolution of Chronic Hypertension Among Pregnant Women in Abidjan’s Neighborhood

**DOI:** 10.3389/ijph.2025.1608442

**Published:** 2025-12-04

**Authors:** Koussoh Simone Malik, Marie Laure Tiade, Guanga David Meless, Franck Renaud Djedro Meless, Jerome Kouame, Julie Sackou-Kouakou, Kassi Anicet Adoubi, Agbaya Serge Oga, Odile Ake

**Affiliations:** 1 National Institute of Public Health (INSP), Abidjan, Côte d’Ivoire; 2 Félix Houphouët-Boigny University, Abidjan, Lagunes, Côte d’Ivoire; 3 Alassane Ouattara University, Bouaké, Vallee du Bandama, Côte d’Ivoire

**Keywords:** hypertension, pregnancy, maternal health, risk factors, blood pressure trends

## Abstract

**Objective:**

In Côte d’Ivoire, prevalence of hypertension was 39.9% in 2017. This study aimed to determine the prevalence, associate health conditions and evolution of chronic hypertension among pregnant women in Abidjan’s neighborhood.

**Methods:**

This was a prospective multicenter cohort study. Bivariate and multivariate analysis was performed.

**Results:**

Prevalence of chronic hypertension was 8.6% [6.11–11.09]. Age (p = 0.001; 95% CI: 2.2–14.5); hyperglycemia (p = 0.04; 95% CI: 1.1–33.5); total cholesterol level (p = 0.02; 95% CI: 1.2–7.3); LDL cholesterol level (p = 0.01; 95% CI: 1.2–5.0); left ventricular hypertrophy (p = 0.03; 95% CI: 1.1–6.6) were significantly associated with chronic hypertension. After multivariate analysis, age ≥35 years (OR = 3.9; 95% CI: 1.4–11.8; p = 0.01) was the only variable statistically associated with chronic hypertension. During the follow-up to full-term pregnancy, systolic and diastolic blood pressures were significantly lower than those collected at inclusion. No complication such as preeclampsia was observed.

**Conclusion:**

The prevalence of chronic hypertension was relatively high. Women aged 35 and over were most at risk of developing the disease. Blood pressure gradually fell and stabilized in the women. No complication was observed.

## Introduction

Hypertension is the leading preventable cause of disease and premature death worldwide [1]. It is quantitatively the most important risk factor for cardiovascular disease [2]. Pregnant women are particularly vulnerable to this disease. Indeed, hypertensive disorders during pregnancy are common complications of pregnancy; these disorders are the second leading cause of pregnancy-related mortality worldwide [3, 4], and are significantly associated with early or late delivery and prenatal morbimortality [5].

Low- and middle-income countries are particularly concerned, with 87% of maternal deaths worldwide by 2020 represented in sub-Saharan Africa and South Asia [6]. In these countries, hypertensive disorders of pregnancy are responsible for around 25% of maternal deaths [4]. Chronic hypertension in pregnancy is identified before pregnancy or before 20 weeks of amenorrhea. It is not only associated with serious maternal complications, such as myocardial infarction, stroke, pre-eclampsia and caesarean section [1, 7], but also with an approximately 5-fold higher risk of maternal mortality and a 2-fold higher risk of neonatal death [8].

In some countries, such as the USA, the prevalence of chronic hypertension is rising regularly, from 1.8% to 3.7% between 2008 and 2021 [9]. In France, 1.5% of nulliparous women and 1.8% of multiparous women had chronic hypertension between 2010 and 2018 [10]. In Cameroon, Amougou et al. reported a prevalence of chronic hypertension of 23.53% among pregnant women with preeclampsia [11]. As for factors associated with the disease, a 2009 study in Brazil revealed that being older, black and obese were important and statistically significant risk factors for chronic hypertension during pregnancy [12].

In 2015, the WHO set targets to be achieved by 2030, including reducing maternal mortality and deaths in newborns and children under 5 years of age [6]. Understanding the factors associated with chronic hypertension would also help reduce maternal mortality. In Côte d'Ivoire, cardiovascular disease is a public health problem, with a mortality rate of 11% [13] and a prevalence of 39.9% for arterial hypertension in 2017 [14]. All causes combined, the maternal mortality ratio remains high, at 480 maternal deaths per 100,000 live births among women of childbearing age (15–49) in the country [15]. However, hypertensive disorders in pregnancy remain poorly documented, and there is very little data on the factors associated with these disorders in pregnant women. The aim of the study was therefore to determine the prevalence, associated factors and evolution of chronic hypertension in a population of pregnant women in Abidjan.

## Methods

### Study Design and Setting

This was a multicenter prospective cohort study among pregnant women in 5 health facilities of the Abobo East health district located in the commune of Abobo (Abidjan, Côte d’Ivoire). The cohort study starts with the recruitment of pregnant women. These women were followed every month until 3 months after delivery. The survey ran from February 2022 to May 2023.

The study was carried out in the commune of Abobo. The study site is located in one of the neighbourhoods of the Abobo commune called Abobo-baoulé, and its total population was estimated at 743,666 inhabitants in 2022 according to the Direction de Coordination du Programme élargi de vaccination (DCPEV) of the Republic of Côte d'Ivoire. The commune of Abobo is located in Abidjan, the economic capital of Côte d’Ivoire. Abobo is Abidjan’s second most populous commune, with numerous neighborhoods, including 19 precarious neighborhoods [16]. Most of these neighborhoods are unserviced, with a growing population and increasing need for housing. Around 60% of the communal population lives in these precarious neighborhoods [17]. The Abobo Est health district was chosen because more than the half of the population lives in precarious conditions.

### Health Facility Selection Criteria

Facilities were selected on the basis of two criteria: the number of first prenatal consultations recorded during the 1st half of 2021 and the presence of a delivery room.

We identified seven first-contact healthcare facilities, each recording between 379 and 552 first prenatal consultations. Health facilities without a delivery room were excluded from the project. We therefore selected 5 health facilities for the project.

### Study Population

The study population consisted of pregnant women seen for consultations in the selected first-contact health facilities.

#### Inclusion and Non-Inclusion Criteria

The study included pregnant women whose gestational age was less than 20 weeks of amenorrhea, whose administrative age was greater than or equal to 18 years, who had been living in the commune of Abobo for at least 6 months and who had signed an informed consent form for the study.

Pregnant women with a pathology affecting their physical and mental ability to answer the questionnaire.

### Sampling

#### Calculation of Sample Size

The sample size was calculated to estimate a proportion of hypertensive disorders of pregnancy of 10% [18] with a risk α of 5% and a precision of 3% using the formula n = z^2^ x p (1 - p)/i^2^

n = sample size; z = 95% confidence level, z = 1.96.

p = estimated proportion of hypertensive disorders in pregnancy. P = 10%.

i = tolerated margin of error or precision of 0.03.

$$n=1.96\;X\;1.96\;X\;0.1\;X\;0.9/\;\left(0.03\right)2=384.16$$

n0 is the initial sample size from which size n is drawn. The expected proportion of women with chronic hypertension is 10%.

n0=n/0.9=385/0.9=427.77 or 428

The minimum sample size is 428, i.e., a minimum of 86 women to be recruited per health facility.

#### Sampling and Data Collection Procedures

Recruitment was systematic. The study was offered to all pregnant women attending their first prenatal consultation at the selected facilities, who were less than 20 weeks’ amenorrhea and who met the inclusion criteria, until the calculated sample size was reached.

Pregnant women were followed up on a monthly basis. The research team consisted of investigators, midwives, biologists and physicians. All investigators underwent a standardized two-day training course on measurement techniques, followed by a practical assessment. The research team was responsible for calling pregnant women to remind them of their appointments. During the visits, data on their state of health were collected, followed by blood pressure measurements.

The frequency of visits was monthly, and the total number of visits was 8 if the women were regular attenders. Visits ended 3 months after delivery.

Several data were collected on inclusion of pregnant women, including sociodemographic data (administrative age in years and education level), gyneco-obstetrical data (parity), anthropometric and clinical data (weight, height, blood pressure and heart rate). Weight was measured on a SECA 750 scale capable of weighing up to 150 kg on a lightly clothed woman. Height was recorded on the identity document. Obesity was assessed by body mass index in the first trimester of pregnancy [19, 20]. Blood pressure was measured in a seated subject after 15 min’ rest, using an OMRON M^6^ automatic electronic sphygmomanometer fitted with a cuff (belonging to the list of sphygmomanometers validated by the French Hypertension Society) [21]. It was taken on the bare arm, placed on a table, palm upwards. Three measurements were taken at 5-minute intervals; the average of the last two measurements was taken for each participant. The highest blood pressure was used. Hypertension was defined as PAS/PAD ≥140/90 and/or women knowing their hypertension status. Heart rate (HR) was measured using an OMRON M^6^ automatic electronic sphygmomanometer.

Biological data (glycemia, creatinine, total cholesterol, HDL cholesterol, LDL cholesterol, triglycerides, uricemia, natremia, kalemia, chloremia, calcemia, magnesemia) and electrocardiographic data (left ventricular hypertrophy) were collected. The electrocardiogram was performed after the blood pressure was taken in a supine position on the examination table. The twelve-lead electrocardiogram was performed on a BIOCARE model iE3 electrocardiograph. Electrocardiographic leads were placed according to standard practice. Recordings were made at 25 mm/s, calibrated at 10 mm/mV and printed on graph paper with time on the x-axis and voltage on the y-axis. Left ventricular hypertrophy (LVH) was objectified by the Sokolow-Lyon index [22] or the Cornell index [23].

During follow-up, blood pressure data were collected. The primary outcome was the evolution of chronic hypertension, characterized by the mean blood pressure of hypertensive women at each visit.

The use of prescription antihypertensives by hypertensive pregnant women was systematic in our study when the woman was newly diagnosed and her blood pressure was greater than or equal to 150/95 mmHg. When blood pressure was greater than or equal to 140/90 mmHg but less than 150/95 mmHg, pregnant women were advised to take dietary hygiene measures. These measures mainly consisted in reducing salt intake. Women already on antihypertensive treatment kept their treatment if it was a drug recommended during pregnancy. For women already taking a non-recommended antihypertensive drug, the initial treatment was stopped and replaced either by alpha methyl dopa monotherapy, or by bitherapy including nicardipine, depending on blood pressure levels. The dosage of alpha methyl dopa varied from 250 to 750 mg, that of nicardipine from 50 to 100 mg. All women had received aspirin 100 mg for the prevention of preeclampsia.

### Statistical Analysis

Data were entered using KoboCollect software and analyzed using R version 4.1.1 and SPSS version 29.0.

Analysis of inclusion data covered sociodemographic, gyneco-obstetrical, anthropometric, clinical, biological and electrocardiographic characteristics. Quantitative variables were expressed as mean ± standard deviation, while qualitative variables were presented as numbers and percentages.

The search for factors associated with chronic hypertension was carried out in several stages. First, a bivariate analysis was performed using Pearson’s Chi-square test, or Fisher’s exact test when the conditions for applying Pearson’s Chi-square were not met. Variables with a p-value of less than 20% were selected for multivariate analysis. Next, a step-by-step top-down selection procedure, based on the Akaike criterion, was used to identify the explanatory variables to be included in the multivariate logistic regression model. The significance level was set at 0.05.

Analysis of follow-up data focused on the evolution of women’s mean blood pressure during follow-up. A linear mixed model with random intercept, followed by an ANOVA, was used to test the overall significance of changes in mean arterial pressure at different gestational ages, with an alpha risk of 5%. In the event of significance, Tukey’s adjustment was applied to make pairwise comparisons between blood pressure at different follow-up periods and that measured at inclusion.

### Ethical Consideration

This study was approved by the Comité National d’Ethique des Sciences de Vie et de la Santé de Côte d’Ivoire. The date of approval was September 2022 with the reference number “Ref: 116-22/MSHPCMU/CNESVS-kp.” Pregnant women were included in the study only when they gave their consent. Study participants were free to withdraw from the study at any time without prejudice. All information about the participants was kept confidential and was known only to the research team. An individual identification number was used for the medical file, data entry and biological data collection.

## Results

### Characteristics of Study Participants

The mean age of the women was 27.4 ± 6.3 years, with a maximum age of 46 and a minimum age of 18. More than a third of the women were between 18 and 24 years old, and 33.3% had secondary school education. Around two out of five women (38.7%) were nulliparous. The mean body mass index (BMI) was 25.7 kg/m^2^ ± 5.3 kg/m^2^. Over seventeen percent of women were obese. Tachycardia was found in 11.6% and 10% of women had left ventricular hypertrophy.

### Prevalence of Chronic Hypertension in Pregnant Women

Forty-two women with had chronic hypertension i.e., a prevalence of 8.6% [95% CI; 6.11–11.09]. The mean age was 34.1 ± 6.5 years. Of these, 23 with a mean age of 34.1 ± 6.5 years were already aware of their hypertensive status, while in 19 women, mean age 28.2 ± 6.4 years, hypertension was discovered during routine screening of women. Women aware of their hypertensive status were significantly older than those discovered at inclusion (p = 0.005).

### Factors Associated With Chronic Hypertension in Pregnant Women

After bivariate analysis, a statistically significant association was observed between chronic hypertension and age, levels of fasting glycemia, total cholesterol level, LDL cholesterol level and left ventricular hypertrophy. Pregnant women aged 35 and over were more than 5 times more likely to have chronic hypertension than those aged 18 to 24 [OR = 5.6; 95% CI: 2.2–14.5; p < 0.001]. Women with hyperglycemia were more than 6 times more likely to have chronic hypertension than those with blood glucose levels below 1.10 g/L [OR = 6.3; 95% CI: 1.1–33.5; p = 0.04]. Participants with total cholesterol levels above 2.5 g/L were 3 times more likely to have chronic hypertension than those with levels below 2.5 g/L [OR = 3.1; 95% CI: 1.2–7.3; p = 0.02]; those with LDL cholesterol levels above 1.3 g/L were 2.4 times more likely to have chronic hypertension than gestational females with LDL cholesterol levels below 1.3 g/L [OR = 2.4; 95% CI: 1.2–5.0; p = 0.01]. Pregnant women with left ventricular hypertrophy were approximately 3 times more likely to have chronic arterial hypertension than those without left ventricular hypertrophy [OR = 2.7; 95% CI: 1.1–6.6; p = 0.03]. Table 1 shows the characteristics of pregnant women at inclusion and their association with chronic hypertension.

**TABLE 1:** Characteristics of pregnant women at inclusion (N = 487) and association with arterial hypertension; Prevalence, Associate Health Conditions and Evolution of Chronic Hypertension Among Pregnant Women in Abidjan’s Neighborhood, Abobo, Côte d’Ivoire, 2022-2023.

Variables	Frequency n (%)	Hypertension no n (%)	Hypertension yes n (%)	OR	95% CI	p-value
Age (years)						0.001*
18–24	178 (36.5)	171 (96.1)	7 (3.9)	-	-	
25–29	143 (29.4)	132 (92.3)	11 (7.7)	2.0	[0.77–5.39]	
30–34	86 (17.7)	77 (89.5)	9 (10.5)	2.9	[1.03–7.95]	
35–46	80 (16.4)	65 (81.3)	15 (18.8)	5.6	[2.20–14.45]	
Education level						*0.59*
Not in school	127 (27.4)	117 (92.1)	10 (7.9)	-	-	
Primary	111 (23.9)	98 (88.3)	13 (11.7)	1.5	[0.7–3.8]	
Secondary	154 (33.2)	143 (92.9)	11 (7.1)	0.9	[0.4–2.2]	
Superior	72 (15.5)	65 (90.3)	7 (9.7)	1.3	[0.4–3.4]	
Parity						*0.42*
Nulliparous	175 (38.7)	162 (92.6)	13 (7.4)	-	-	
Primiparous	121 (26.8)	110 (90.9)	11 (9.1)	1.3	[0.5–2.9]	
Multiparous	156 (34.5)	138 (81.8)	18 (18.2)	1.6	[0.8–3.5]	
Obesity						*0.46*
Obesity yes	58 (17.7)	51 (87.9)	7 (12.1)	-	-	
Obesity no	270 (82.3)	246 (91.1)	24 (8.9)	1.4	[0.5–3.3]	
HR^a^ (beats/minute)						*0.09*
HR^a^ ≤100	380 (88.4)	352 (92.6)	28 (7.4)	-	-	
HR^a^ >100	50 (11.6)	43 (86.0)	7 (14.0)	2.1	[0.8–4.7]	
Glycemia (g/L)						0.04*
Glycemia <1.10	434 (97.1)	402 (92.6)	32 (7.4)	-	-	
1.10 ≤glycemia <1.26	7 (1.6)	6 (85.7)	1 (14.3)	2.1	[0.1–12.8]	
Glycemia ≥1.26	6 (1.3)	4 (66.7)	2 (33.3)	6.3	[1.1–33.5]	
TC^b^ (g/L)						0.02*
TC^b^ ≤2.5	409 (91.5)	381 (93.2)	28 (6.8)	-	-	
TC^b^ >2.5	38 (8.5)	31 (81.2)	7 (18.4)	3.1	[1.2–7.3]	
HDL-C (g/L)						0.15
HDL-C <0.30	9 (2.0)	7 (77.8)	2 (22.2)	-	_	
HDL-C ≥0.30	438 (98.0)	405 (92.5)	33 (7.5)	0.3	[0.1–1.9]	
LDL-C (g/L)						0.01*
LDL -C ≤1.30	282 (63.1)	267 (9 4.7)	15 (5,3)	-	-	
LDL-C >1.30	165 (36.9)	145 (88.0)	20 (12.0)	2.4	[1.2–5.0]	
Triglyceride level (g/L)						*0.70*
TG^c^ ≤1.20	360 (86.3)	333 (92.5)	27 (7.5)	-	-	
TG^c^ >1.20	57 (13.7)	52 (91.2)	5 (8.8)	1.2	[0.4–3.0]	
Uricemia (mg/L)						*0.70*
Uricemia ≤70	437 (99.1)	403 (92.2)	34 (7.8)	-	-	
Uricemia >70	4 (0.9)	4 (100.0)	0 (0.0)	0.001	[0.1–1.5]	
Natremia (mEq/L)						*0.09*
Natremia <135	7 (1.6)	5 (71.4)	2 (28.6)	-	-	
Natremia ≥135	440 (98.4))	407 (92.5)	33 (7.5)	0.2	[0.1–1.5]	
Kaliemia (mEq/L)						*0.41*
Kaliemia <3.6	26 (5.8)	26 (100)	0 (0)	-	-	
3.6 ≤Kaliemia <5	418 (93.5)	383 (91.6)	35 (8.4)	2.0	[0.8–33.5]	
Kaliemia ≥5	3 (0.7)	3 (100)	0 (0)	1.0	[0.1–15.8]	
Magnesemia (mg/L)						*0.18*
Magnesemia <17	142 ()	131 (92.3)	11 (7.7)	-		
Magnesemia (17–25)	298 ()	276 (92.6)	22 (7.4)	1.0	[0.5–2.1]	
Magnesemia >25	8 ()	6 (75.0)	2 (25.0)	4.0	[0.5–19.8]	
Calcemia (mg/L)						*0.70*
Calcemia ≤110	431 ()	397 (92.1)	34 (7.9)	-	-	
Calcemia >110	17 ()	16 (94.1)	1 (5.9)	[0.7]	[0.1–3.7]	
Chloremia (mg/L)						*0.15*
Chloremia ≤105	436 (99.5)	403 (92.4)	33 (7.6)	-	-	
Chloremia >105	2 (0.5)	1 (50.0)	1 (50.0)	12.2	[0.5–3.1]	
LVH^d^						0.03*
LVH^d^ no	342 (90.0)	316 (92.4)	26 (7.6)	-	-	
LVH^d^ yes	38 (10.0)	31 (81.6)	7 (18.4)	2.7	[1.1–6.6]	

^a^ HR, heart rate.

^b^ TC, total cholesterol.

^c^ TG, triglyceride level.

^d^ LVH, left ventricular hypertrophy; OR, Odd ratio. CI, Confidence interval

The bold values represents the statistically significant p-values (p < 0.05).

After multivariate analysis (Table 2), at constant levels of other variables, age was the only variable statistically associated with chronic hypertension in pregnant women. Pregnant women aged 35 and over were approximately 4 times more likely to be hypertensive [OR = 3.9; 95% CI:1.4–11.8; p = 0.01].

**TABLE 2:** Multivariate analysis, logistic regression of factors associated with chronic hypertension; Prevalence, Associate Health Conditions and Evolution of Chronic Hypertension Among Pregnant Women in Abidjan’s Neighborhood, Abobo, Côte d’Ivoire, 2022-2023.

Variables	Ajusted OR	95% CI	p-value
Age			0.01*
18–24	-	-	
25–29	2.2	[0.8–6.6]	
30–34	1.8	[0.5–6.2]	
35–46	3.9	[1.4–11.8]	
Glycemia			*0.1*
<1.10	-	-	
1.1 ≤ Blood sugar <1.26	1.7	[0.1–10.9]	
≥1.26	5.0	[0.6–31.8]	
Total cholesterol			*0.4*
≤2.5	-		
>2.5	1.6	[0.5–4.3]	
LDL cholesterol			**0.06**
≤1.30	-	-	
>1.3	2.1	[1.0–4.7]	

The bold values represents the statistically significant p-values (p < 0.05).

### Evolution of Chronic Hypertension

In our study, 9 women (21.4%) received dietary hygiene measures and aspirin as treatment, 28 women (66.7%) received monotherapy, aspirin and dietary hygiene measures; and 5 women (11.9%) received bitherapy, aspirin and dietary hygiene measures.

**FIGURE 1:**
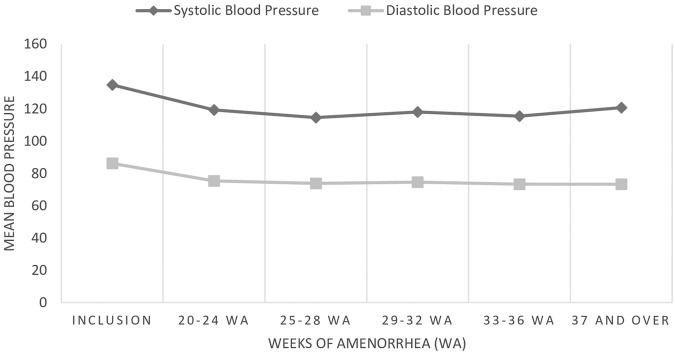
Evolution of blood pressure in pregnant women with chronic hypertension (N = 42); Prevalence, Associate Health Conditions and Evolution of Chronic Hypertension Among Pregnant Women in Abidjan’s Neighborhood, Abobo, Côte d’Ivoire, 2022-2023.

The evolution of the mean blood pressure of hypertensive pregnant women from inclusion to the end of pregnancy was therefore quite good overall (Figure 1).

The mean blood pressure at inclusion was 134.83 mmHg for systolic blood pressure and 86.19 mmHg for diastolic blood pressure. During the follow-up to full-term pregnancy, systolic and diastolic blood pressures were significantly lower than those at baseline (Table 3).

**TABLE 3:** Comparison of blood pressures in chronically hypertensive pregnant women at inclusion and at different weeks of amenorrhea; Abobo, Côte d’Ivoire (N = 42), Prevalence, Associate Health Conditions and Evolution of Chronic Hypertension Among Pregnant Women in Abidjan’s Neighborhood, Abobo, Côte d’Ivoire, 2022-2023.

Weeks of amenorrhea	SBP^a^ (mmHg)		DBP^b^ (mmHg)	
	Mean BP	SD^d^	Mean	SD
At inclusion	134.8	22.3	86.2	14.6
Between 20 and 24 WA^d^	119.3	19.2	75.4	15.2
p-value*	***0.0026***	-	***0.0013***	-
Between 25 and 28 WA	114.6	17.1	73.8	15.1
p-value*	***0.0001***	-	***0.0033***	-
Between 29 and 32 WA	118.0	14.7	74.5	12.0
p-value*	***0.0153***	-	***0.0037***	-
Between 33 and 36 WA	115.5	16.4	73.3	14.4
p-value*	***<0.0001***	-	***0.0003***	-
37 WA and over	120.7	16.8	73.4	13.8
p-value*	*0.0691*		*0.0978*	

^a^ SBP, systolic blood pressure.

^b^ DBP, diastolic blood pressure.

^c^ WA, Weeks of amenorrhea.

^d^ SD, Standard deviation.

*p-value SBP or DBP difference: statistically significant difference vs. PA’s inclusion (Reference).

The bold values represents the statistically significant p-values (p < 0.05).

Blood pressure gradually fell and stabilized in the women, with mean systolic/diastolic values rising from 119.28/75.36 mmHg between 20 and 24 weeks’ amenorrhea (WA) to 115.46/73.33 mmHg between 33 and 36 SA (Figure 1). On the other hand, at 37 or more WA, blood pressure increased slightly but remained at a mean value not significantly different from the value at inclusion (Table 3). We had no complications such as preeclampsia.

## Discussion

The aim of this study was to determine the prevalence, associated factors and evolution of chronic hypertension in a population of pregnant women with a mean age of 27.4 ± 6.3 years in the Abobo East health district.

We noted a prevalence of chronic hypertension of 8.6% among pregnant women whose mean age was 34.1 ± 6.5 years. A number of factors associated with chronic hypertension in pregnant women in the bivariate analysis were no longer associated with chronic hypertension after multivariate analysis in our study. These factors were hyperglycemia, total cholesterol level, LDL cholesterol level and left ventricular hypertrophy.

The independent factor associated with chronic hypertension was age greater than or equal to 35 years. Women aged 35 years and older were four times more likely to have chronic hypertension than those aged 18–24 years.

The prevalence of chronic hypertension in our investigation is superposable to that noted by Gudnadottir et al. in Iceland, which was 9% [19]. The prevalence of chronic hypertension in pregnant women in our series is lower than that noted by authors in Ghana and Lesotho [24, 25], as well as that reported by authors in the USA in a population of non-Hispanic black women [26, 27]. In these investigations, the prevalence of chronic hypertension ranged from 10.2% to 19%. On the other hand, the prevalence of chronic hypertension in our population is higher than that reported by other authors in Africa [28, 29] and outside Africa [30]. The prevalence expressed by these authors varied from 1.9% to 2.7%. These differences could be explained by the origin of the population, by their lifestyles, which could be different from ours, by the large size of the population used for the study in China, unlike Ghana, which had a population almost similar to ours (500 pregnant women), or by the method of investigation (some authors carried out case-control studies or studies based on the birth population).

The prevalence of high blood pressure is relatively high in our population, which is a young population. Emphasis must be placed on preventing risk factors for chronic hypertension. It is important to implement awareness programs on the risks of hypertension during pregnancy, targeting women of childbearing age and their families. This could include community -based workshops and media campaigns.

A number of factors associated with chronic hypertension. Concerning hyperglycemia, we did not establish that the women with hyperglycemia in our survey had diabetes mellitus, diabetes mellitus being a chronic metabolic disease characterized by persistent hyperglycemia [31]. However, studies in Africa [31–33] and a study in Taiwan have demonstrated a positive association between diabetes mellitus and chronic hypertension. Similarly, according to the study by Mengesha et al. in Botswana, people with type 2 diabetes had a significant risk of hypertension [32]. In our investigation, there was a significant association between high total cholesterol as well as high LDL cholesterol and chronic hypertension. Adeniyi et al. also noted in their study that individuals with high total cholesterol levels were about twice as likely to be hypertensive [31]. In the USA, Egan et al. analyzed reports from the National Health and Nutrition Examination Surveys (NHANES) and concluded that 60.7%–64.3% of hypertensive patients also suffered from hypercholesterolemia [34]. Indeed, hypercholesterolemia is associated with increased secretion of vasoconstrictor molecules or vasoactive substances and is responsible for arterial stiffness [35, 36]. Our research has also demonstrated a significant association between chronic hypertension and left ventricular hypertrophy. These findings are consistent with those of Dasai et al., who found that individuals with electrocardiographic left ventricular hypertrophy were more likely to be black and to have higher systolic blood pressure [37]. Left ventricular hypertrophy occurs as a pathophysiological adaptation to increased chronic afterload and serves as an independent predictor of cardiovascular events [38].

The independent factor associated with chronic hypertension in our study was age. Around 20% of women aged 35 and over were hypertensive, and the difference was significant; these women were 4 times more likely to have chronic hypertension than those aged 18 to 24. These results are in line with studies in Africa [29] and outside Africa [30, 39], which have shown that women aged 35 and over have a higher risk of hypertensive disorders of pregnancy than younger women. Thus, advanced age has been associated with an increased incidence of chronic hypertension, because various physiological changes, including atherosclerosis and vascular changes occurring with aging, are responsible for most cases of chronic hypertension in older populations [40].

Women diagnosed as hypertensive were put on treatment with either dietary hygiene measures and aspirin if blood pressure was below 150/95 mmHg or antihypertensive treatment, dietary hygiene measures and aspirin [41]. We observed a reduction in blood pressure during follow-up compared with inclusion. Therapeutic management coupled with dietary hygiene measures helped control the women’s chronic hypertension. Similarly, a meta-analysis had revealed that in women receiving antihypertensive treatment, compared with those receiving placebo or no treatment, mean blood pressure after inclusion in the trials was significantly lower (mean difference −4.2 mmHg; 95% CI: −6.6 −1.8; p = 0.006) [7]. Indeed, pregnant women with blood pressure elevations ≥150/95 mmHg should be treated with antihypertensive therapy [41]. The goal of treating hypertension during pregnancy is to protect the woman from dangerously high blood pressure, thereby reducing the maternal risk and allowing the pregnancy to continue and the fetus to grow and mature [41, 42]. In the last trimester of pregnancy, blood pressure increased in these women to a value comparable to that recorded at the time of their inclusion in the study. This observation was in line with the literature, since in the normal physiology of pregnancy, blood pressure falls physiologically during the 1st trimester of pregnancy, is stable during the 2nd trimester and then rises to its previous level during the 3rd trimester of pregnancy [43–45]. Continuous monitoring of blood pressure in hypertensive pregnant women is therefore also useful after delivery.

### Study Limitation

One of the study limitations was the pregnant women lost to follow-up. The loss to follow-up rate was 23.4%. Those lost to follow-up may be a source of selection bias if they differ from the women followed until the end of the study. The analyses were performed on the participants who were followed up, and the baseline characteristics of the participants who were lost to follow-up did not differ significantly from those who remained, thus minimizing potential bias. This reduced the sample size at several levels of statistical analysis. However, calculating the sample size by taking into account possible non-responses helped to minimize this bias. The study was conducted only in the Abobo East district, which may not be representative of the Abobo commune and even less of the city of Abidjan. We did not include other factors such as salt consumption, diet, socio-economic level which are factors associated with high blood pressure. Treatment adherence was only assessed by the question “did you take your medication correctly all the time?”

### Conclusion

The prevalence of chronic hypertension among pregnant women was relatively high in urban areas. Women aged 35 and over were most at risk of developing the disease. The study also showed that regular clinical and therapeutic follow-up of chronic hypertensive pregnant women favored blood pressure control throughout pregnancy. Hence the importance of raising pregnant women’s awareness of the need for early detection of hypertension and rigorous medical monitoring of pregnancy.
